# Risk Factors for Progression of Cervical Congenital Scoliosis and Associated Compensatory Curve Behavior

**DOI:** 10.3390/jcm13113039

**Published:** 2024-05-22

**Authors:** Amir A. Amanullah, Taemin Oh, Brandon J. Toll, Akul Patel, Amer F. Samdani, Joshua M. Pahys, Andrew Jeongyoon Kim, Aniketh Vellanki, Jessica Steindler, Terrence G. Ishmael, Steven W. Hwang

**Affiliations:** 1 Lewis Katz School of Medicine at Temple University, Philadelphia, PA 19140, USA; amanuaam97@gmail.com; 2 Shriners Children’s–Philadelphia, Philadelphia, PA 19140, USA; taemin.oh87@gmail.com (T.O.); asamdani@shrinenet.org (A.F.S.); jpahys@shrinenet.org (J.M.P.); andrewkim911@gmail.com (A.J.K.); anikethv14@gmail.com (A.V.); jessica.steindler@shrinenet.org (J.S.); terrence.ishmael@shrinenet.org (T.G.I.); 3 Department of Orthopaedic Surgery and Sports Medicine, Temple University Hospital, Philadelphia, PA 19140, USA; akul.patel@tuhs.temple.edu

**Keywords:** congenital cervical scoliosis, compensatory curve, secondary curve, cervical spine deformity, risk factors, pediatric scoliosis

## Abstract

**Background:** This study investigated risk factors for progression of deformity in pediatric congenital cervical scoliosis (CCS) and evaluated the correlation between congenital cervical curves and compensatory thoracic and lumbar curves. **Methods:** Medical records were retrospectively reviewed for 38 pediatric patients with CCS with a minimum 2-year follow-up. Curve progression was defined as >10° increase in cervical coronal curve angle between presentation and last follow-up. **Results:** A total of 38 patients (16 girls, 22 boys) with a mean age at presentation of 5.6 ± 4.1 years met the inclusion criteria. Sixteen patients (42%) had curve progression with a mean follow-up of 3.1 ± 3.0 years. At presentation, T1 slope was significantly larger among children with progressive deformities (*p* = 0.041). A total of 18 of the 38 patients with strictly cervical spine deformity were then selected for subanalysis to evaluate the progression of compensatory curves. Cervical major coronal curves were found to significantly correlate with lumbar major coronal curves (r = 0.409), C2 central sacral vertical line (CSVL) (r = 0.407), and C7-CSVL (r = 0.403) (*p* < 0.05). Thoracic major coronal curves did not significantly correlate with cervical major coronal curves (r = 0.218) (*p* > 0.05). **Conclusion:** In conclusion, 42% of osseous CCS curves progressed over time in the overall cohort, and high initial T1 slope was found to be most highly correlated with progression of cervical deformity. Cervical major coronal curves significantly correlated with lumbar curve magnitude but not with thoracic curve size in isolated CCS, possibly due to the increased flexibility of the lumbar spine which may allow greater compensatory balance and thus have a greater correlation with cervical curve magnitude and possibly progression.

## 1. Introduction

Congenital scoliosis accounts for approximately 10% of all pediatric scoliosis and encompasses a heterogeneous spectrum of disorders resulting from abnormal vertebral development during early gestational development [1,2,3]. Unlike other causes of scoliosis in children (neuromuscular, syndromic, idiopathic), congenital scoliosis presents unique challenges in presentation and management. Broadly categorized as either failure of formation or failure of segmentation, these anomalies lead to spinal growth disequilibrium and can subsequently lead to spinal deformity [3,4,5]. Many of these conditions are extremely rare, and often coincide with abnormalities in other organ systems, such as in the case of VACTERL and Klippel–Feil syndrome. Even rarer collagenopathies (e.g., osteogenesis imperfecta), skeletal dysplasias (e.g., Larsen syndrome, Goldenhar syndrome), and mucopolysaccharidoses (e.g., Morquio syndrome) can all lead to significant deficiencies in spinal development and growth [6,7]. Spinal deformity in congenital scoliosis most commonly manifests in the thoracolumbar spine, and progression occurs most rapidly in the first 3 years of life [4,5]. Cervical spine involvement is encountered less frequently [8,9,10,11,12], although certain diagnoses such as Klippel–Feil syndrome account for a disproportionate number of cases of congenital cervical scoliosis (CCS) [11].

Optimum management of CCS remains a matter of debate. In thoracolumbar congenital scoliosis, curve progression and failure of bracing are generally considered to be robust indications for surgical intervention [13]. However, it remains unclear if the same operative criteria should be applied for cervical deformity [9,10,11,12,13,14]. Given the unique biomechanics of the cervical spine and the proximity to the cervical spinal cord, CCS presents unique challenges in management, and surgical correction in these patients can carry significant morbidity. Naturally, while proper patient selection plays a significant role in ensuring good outcomes, there is currently limited information within the literature as to which patients would benefit most from surgery. In these patients, the decision to operate must further be balanced against timing of intervention, as earlier intervention offers the benefit of working with a smaller curve, reducing the degree of correction required, and thus improving the risk profile of the surgery.

Proper risk stratification can help shape the management discussion with patients and families. The goal of this present study was to identify which patients may be at higher risk for progression and, by extension, potentially derive greater benefit from operative intervention. We also evaluated the relationship between cervical deformity and compensatory changes in the thoracic and/or lumbar spine, as improving our understanding of the natural history of these curves can further help identify which patients are at risk of progression. Here, we report our institutional experience in managing all patients with CCS.

## 2. Materials and Methods

### 2.1. Study Population

We conducted a retrospective review of all pediatric patients with CCS treated at our institution between January 2008–December 2016. Inclusion criteria were as follows: (1) age ≤ 21 years old; (2) radiographic diagnosis of scoliosis, defined as coronal curve > 10°; (3) presence of congenital vertebral formation or segmentation errors on AP radiographs; and (4) minimum 2-year follow-up. This study is a case series of consecutive patients treated surgically for CCS. There were no specific exclusion criteria. All data were collected under IRB approval (Protocol PHL1713R), and consent for study inclusion was obtained from the patients’ parents.

### 2.2. Data Collection

All data pertaining to patient demographics, radiographic parameters, and follow-up were queried and collected. Among patients who underwent surgery, the date of last follow-up was defined as the last follow-up prior to surgical intervention; otherwise, the most recent clinic follow-up was designated as the last follow-up among the nonoperative cohort. All variables were collected and measured by a single trained individual to ensure measurement consistency. Data were additionally validated by two fellowship-trained pediatric spine surgeons.

Measured radiographic parameters included the following: cervical coronal curve, thoracic coronal curve, lumbar coronal curve, C2 and C7 central sacral vertical line (CSVL; distance between the central sacral plumb line and midpoints of C2 and C7 vertebrae in the coronal plane), C2 and C7 sagittal–vertical axis (SVA; sagittal distance from the sagittal sacral plumb line to the midpoints of the C2 and C7 vertebrae), thoracic kyphosis (TK), lumbar lordosis (LL), T1-slope (T1S), cervical lordosis (CL), O-C2 angle (McGregor Line), C2-T3 angle, and T1-slope minus cervical lordosis (T1S minus CL). Cervical spine measures were collected using standard techniques previously reported in the literature [15]. Curve progression was defined as >10° increase in coronal curve angle.

### 2.3. Statistical Analysis

**Figure 1:**
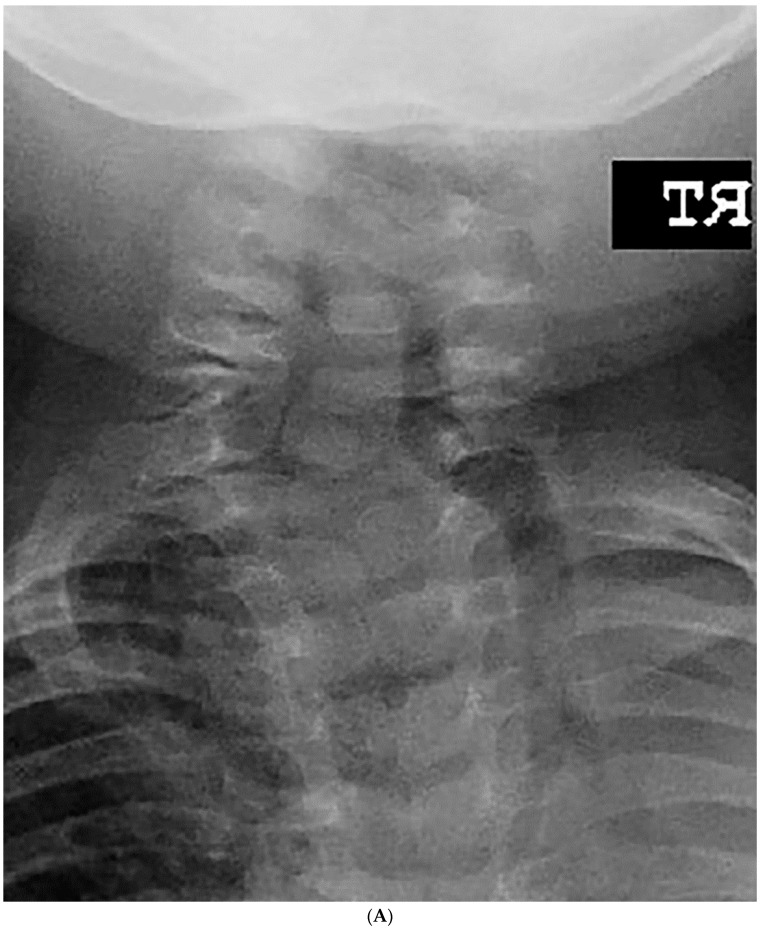

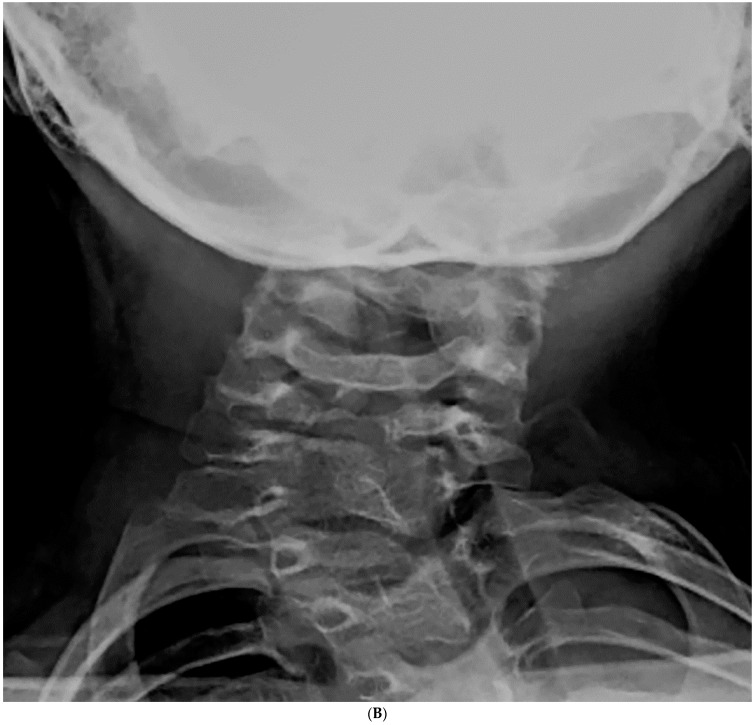

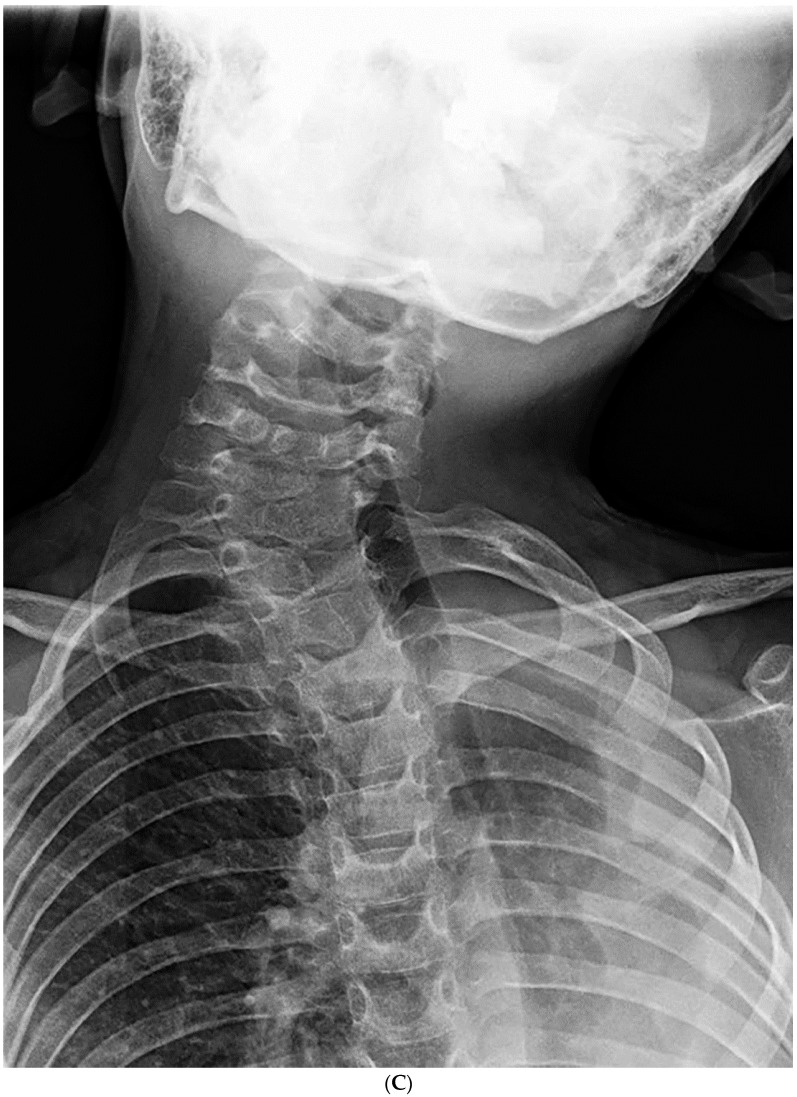
(**A**–**C**). A 10-year-old boy diagnosed with congenital cervical scoliosis (CCS) initially (**A**). This patient has C3 and C7 hemivertebrae with progression of cervical and compensatory curves (**B**,**C**).

**Figure 2:**
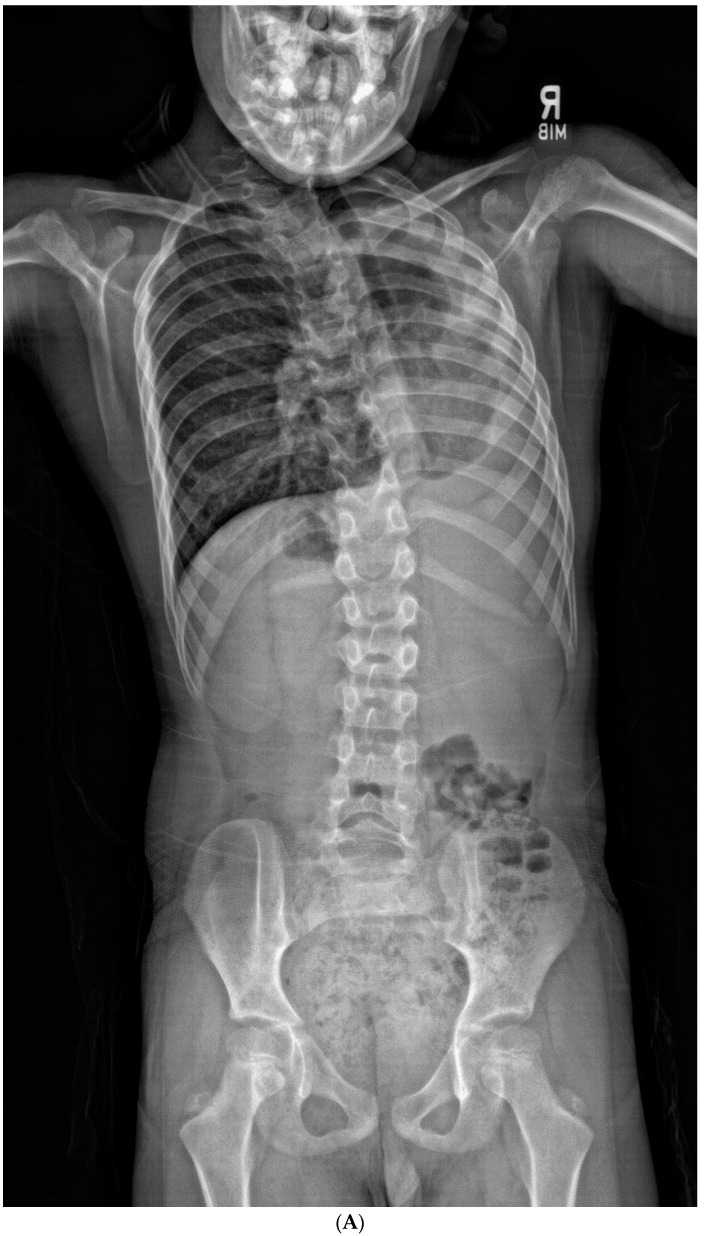

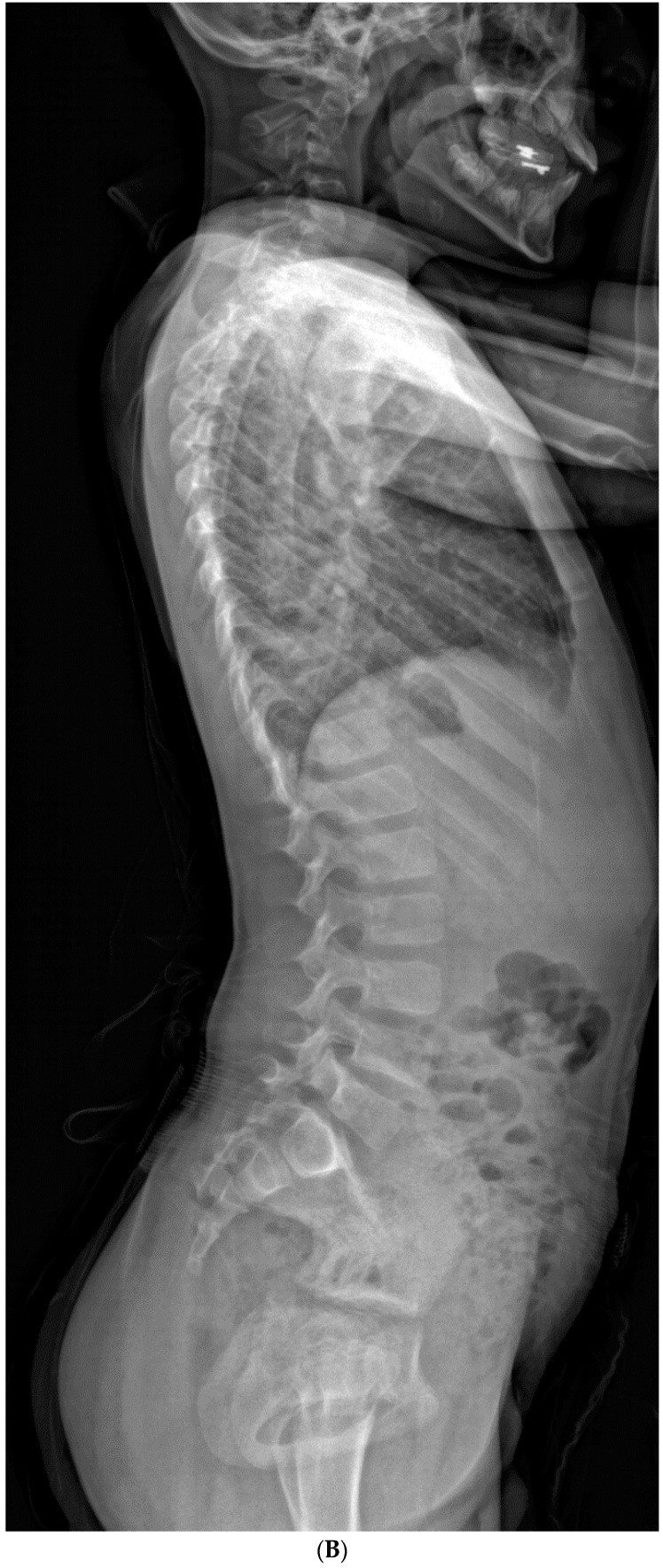

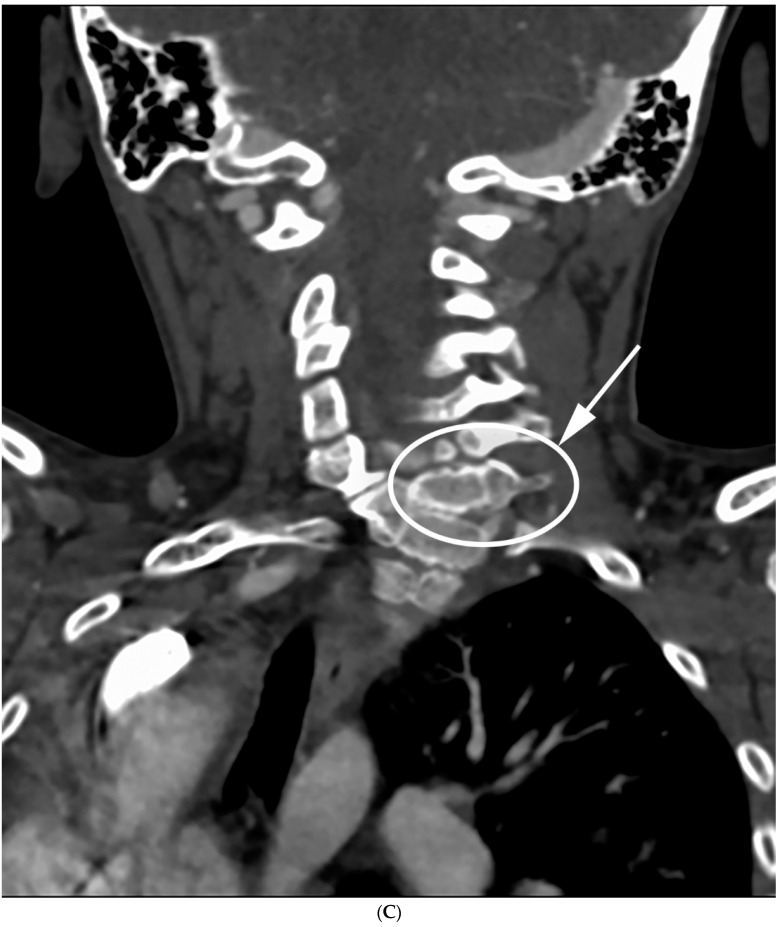
(**A**–**C**). An 8-year-old boy diagnosed with CCS with progression of cervical deformity and compensatory curve. (**A**) PA radiograph shows a C7 hemivertebra. (**B**) Lateral radiograph. (**C**) Coronal CT highlights the left C7 hemivertebra. Patients with thoracic or lumbar vertebral anomalies (such as hemivertebrae, butterfly vertebrae, etc.) were excluded from this study. This was to best ensure that any compensation in the thoracic or lumbar regions was not due to vertebral anomalies in those regions.

IBM SPSS 26.0 was utilized to perform statistical analysis. Mean differences between groups were calculated using student *t*-test, and Cohen’s d was determined for effect size. Pearson correlation test was used to determine linear correlation between variables. Non-independence was accounted for using a 1000-sample bias-corrected bootstrap for the correlation analysis. To evaluate the natural history of how the thoracic and lumbar spines compensate in response to progression of cervical deformity, subgroup analysis was performed on CCS patients without concurrent congenital thoracic or lumbar spine anomalies. Two examples of such patients are shown in Figure 1A–C and Figure 2A–C.

## 3. Results

**Figure 3:**
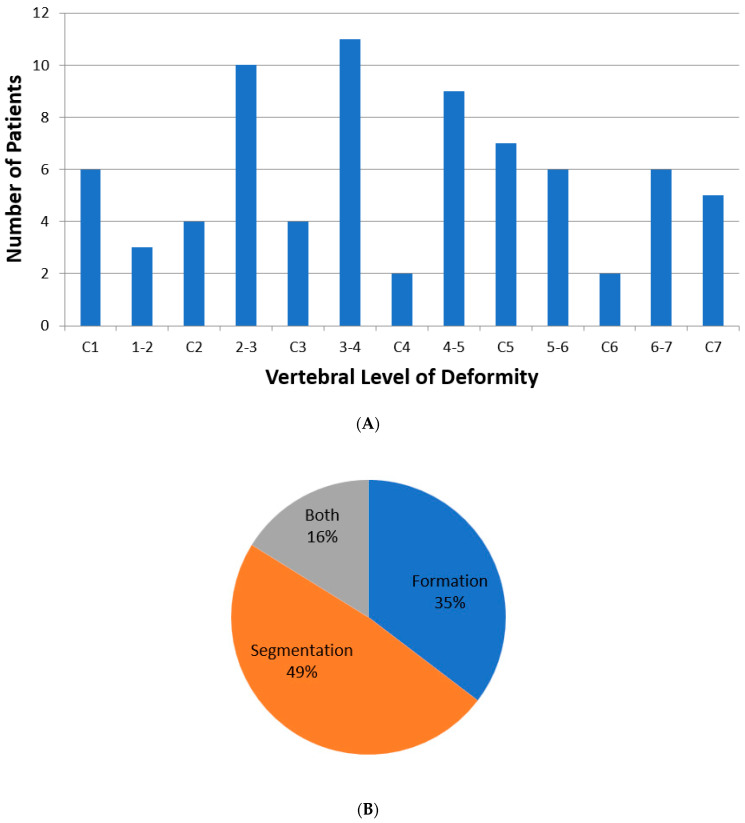
(**A**) Distribution of patients stratified by vertebral level of osseous anomaly. Hyphenated columns represent junctional deformities. (**B**) Pie chart depicting proportions of types of osseous anomalies, characterized as failures of formation, failures of segmentation, or failures of both.

A total of 38 patients (22 male; 16 female) met inclusion criteria for analysis. Mean age at presentation was 5.6 ± 4.1 years, with average follow-up of 3.1 ± 3.0 years. Klippel-Feil syndrome was the most common primary diagnosis (60%), followed by torticollis (21%) (Table 1). All patients had documented osseous anomalies, with a total of 76 total anomalies observed in the cohort. Overall, 48% of patients had segmentation failure, 35% had formation failure, and 16% had both. Figure 3A,B demonstrate the full spectrum of osseous deformities by vertebral level.

**Figure 4:**
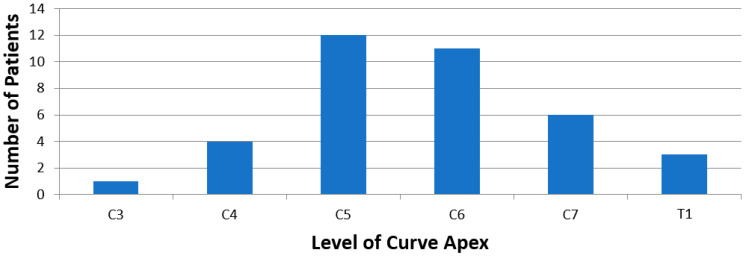
Distribution of patients stratified by vertebral level of apex of deformity.

At time of presentation, mean cervical curve for the entire cohort was 17.6° ± 16.6°, C7-CSVL was 14.7° ± 30.6°, C7-SVA was 35.2° ± 35.3°, and T1S was 27.5° ± 11.3°. As shown in Figure 4, the apex of the curve was most commonly at C5 (32%) and C6 (29%). A total of 16 children (42%) demonstrated curve progression. Patients in the “no progression” versus “progression” groups presented with a similar initial cervical curve (18.6° ± 15.8° vs. 16.3° ± 18.1°, respectively; *p* = 0.682). Over time, both groups of patients demonstrated an increase in mean cervical curve, with the “no progression” cohort increasing by 5.3° ± 4.0° and the “progression” cohort increasing by 8.4° ± 7.2° (Table 2). At time of last follow-up, the “no progression” cohort had smaller cervical curves (22.2° ± 15.8°) compared to the “progression” cohort (26.2° ± 18.4°), although this did not reach statistical significance (*p* = 0.520).

**Figure 5:**
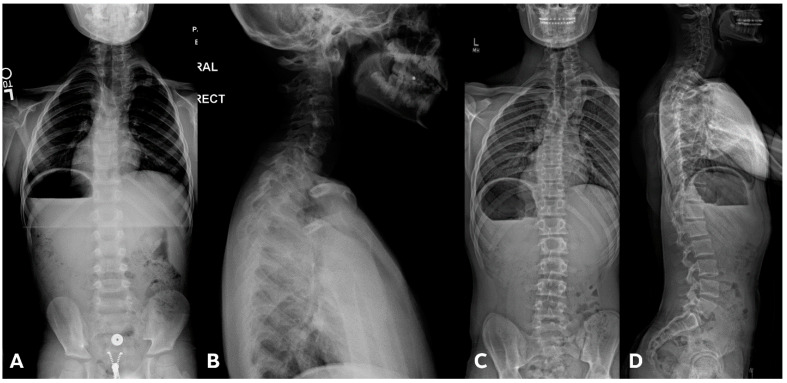
(**A**,**B**) Initial PA and lateral radiograph of a 7-year-old child with a 19° cervicothoracic scoliosis and T1 slope of 31°. (**C**,**D**) Follow-up PA and lateral radiographs showing progression of the curve to 38° over 8 years.

**Figure 6:**
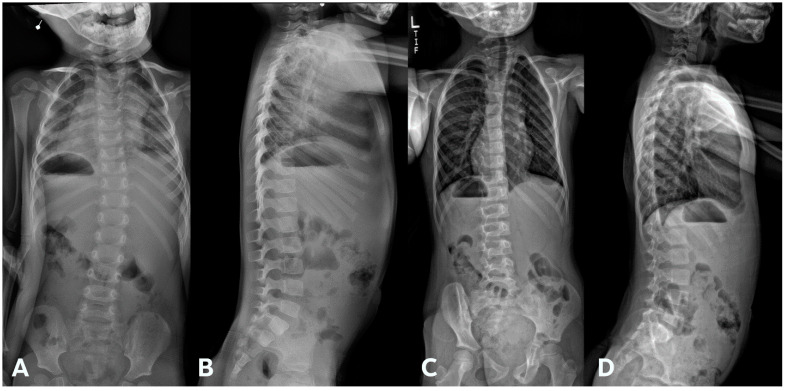
(**A**,**B**) Initial PA and lateral radiograph of a 2-year-old child presenting with 17° of scoliosis at the cervicothoracic junction and T1 slope of 11°. (**C**,**D**) Follow-up radiographs showing a stable curve approximating 18° 4 years later.

Patients in the “progression” cohort presented with a significantly larger T1S compared to the “no progression” cohort (32.4° ± 14.0° vs. 23.8 ± 7.1°, respectively; *p* = 0.041, effect size = 0.78). Individual patients with T1S ≥ 25° were found to be 4.62 times more likely to undergo progression than those who did not (Figure 5A–D and Figure 6A–D). Although these findings mostly trended toward significance, patients in the “progression” cohort also had lower C2-SVA (20.8° ± 30.7° vs. 57.5° ± 41.1°, *p* = 0.053), small thoracic curves (33.2° ± 20.6° vs. 54.1° ± 22.6°, *p* = 0.063), greater LL (59.3° ± 13.2° vs. 45.3° ± 12.1°, *p* = 0.056), and smaller interval change in C2-CSVL (−4.9° ± 21.8° vs. 18.4° ± 23.1°, *p* = 0.069).

**Figure 7:**
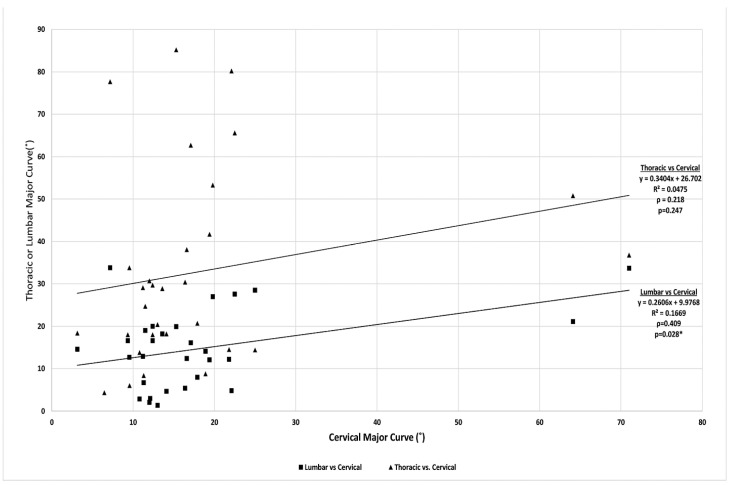
Scatterplot of thoracic or lumbar major coronal curves (y-axis) compared to cervical major coronal curves (x-axis). Thoracic vs. cervical major coronal curves are depicted by the triangles. Lumbar vs. cervical major coronal curve are depicted by the squares. The corresponding Pearson correlation and significance are listed to the right. The asterisk denote no value since the x and y values are the same.

Subgroup analysis was subsequently performed on patients who exclusively had CCS without congenital anomalies in the thoracic or lumbar spine. These 18 patients contributed 40 individual time points for Pearson correlation to determine relationships between cervical coronal curves and the thoracic/lumbar coronal curves, respectively (Figure 7). As shown in Table 3, these patients had a mean cervical curve of 16.0° ± 14.7°, with a compensatory thoracic curve of 33.7° ± 25.6° and compensatory lumbar curve of 14.8° ± 10.0°. Mean TK was 43.5° ± 10.6°, LL was 43.8° ± 16.6°, and CL was −8.1° ± 23.5° (negative value denoting cervical kyphosis). In this patient cohort, the cervical deformity progressed on average 5.4°/year and had a significant increase in cervical curve (16.0° ± 14.7° to 21.7° ± 16.4°, ∆ = 5.7 ± 5.6; *p* = 0.005) and LL (43.8° ± 16.6° to 48.0° ± 17.4°, ∆ = 4.0 ± 3.8; *p* = 0.040) between initial presentation and last follow-up. 

Table 4 depicts Pearson correlation analysis comparing the cervical and thoracic curves to other radiographic parameters. Cervical curves did not correlate with thoracic curves (*p* > 0.05) but positively correlated with the lumbar curve (ρ = 0.409; *p* = 0.028), C2-CSVL (ρ = 0.407; *p* = 0.035), and C7-CSVL (ρ = 0.403; *p* = 0.037). Cervical curves were also found to have a significantly negative correlation with T1S (ρ = −0.344; *p* = 0.034). In comparison, thoracic curves positively correlated with lumbar curve (ρ = 0.377; *p* = 0.048) and cervical lordosis (ρ = 0.408; *p* = 0.028), while negatively correlating with lumbar lordosis (ρ = −0.437; *p* = 0.037) and T1S minus CL (ρ = −0.537; *p* = 0.003).

## 4. Discussion

CCS is a rare but potentially serious condition which poses significant risk to a child’s biomechanical, neurological, and psychosocial well-being. In comparison to the thoracolumbar congenital scoliosis literature, which demonstrates overall high rates of rapid and severe curve progression, there are few studies examining the manifestation of secondary compensatory curves in patients with CCS. This highlights the need to improve our understanding of how cervical deformity impacts the rest of the spine, as the relationship between cervical deformity and secondary radiographic variables remains poorly understood.

Previous reports on congenital scoliosis have consistently identified age, height, and weight as primary longitudinal predictors of deformity progression [2,8,14,15]. While our data set did not reproduce these findings, we believe this is due to our relatively young patient cohort (mean age 5.64 ± 4.12 years) and our 2-year minimum follow-up, which may be insufficient in capturing changes that occur primarily with the onset of puberty. Thus, our study may underestimate the true risk of progression. Longitudinal prospective studies of CCS would better serve the purpose of identifying these potential trends more accurately. From a radiographic perspective, our analysis of the present cohort suggests that children presenting with CCS who have higher T1S are at higher risk for deformity progression compared to those with lower T1S (32.4° ± 14.0° vs. 23.8° ± 7.1°, respectively, *p* = 0.041). This finding nearly reached a large effect size (d = 0.78), and risk calculation revealed that children with T1S ≥ 25° were 4.62 times more likely to undergo deformity progression. This finding is intuitive, as higher T1S suggests loss of cervical lordosis and in some cases even cervical kyphosis [16]. Samartzis et al. [10] identified a relationship between the rigidity of the cervical segment and faster deformity progression, postulating that fused segments in Klippel–Feil syndrome create a fulcrum against adjacent vertebrae that become unable to compensate. In that manner, it may be that a T1S > 25° represents a “tipping point” after which the ability to compensate along the sagittal plane is lost, leading to rapid progression of the deformity. Interestingly, the differences in T1S were not significant when looking at the “no progression” vs. “progression” cohorts at time of last follow-up (25.4 vs. 26.2, respectively; *p* = 0.784). We feel that this finding perhaps reflects the underlying compensatory changes that occur in the thoracic and lumbar spines to balance alignment as these curves progress. It is also worth noting that the T1S and CL mismatch failed to predict risk of progression, despite this having been shown in the adult spine deformity literature to serve as a marker for cervical deformity [17]. While no other radiographic differences reached threshold for significance, children with greater LL also showed a trend towards increased risk of progression (59.32° ± 13.18° vs. 45.34° ± 12.05°, *p* = 0.056). This finding is likely reflective of compensatory changes in the lumbar spine in relation to cervical deformity and thus may serve as an additional marker for indicating severity of cervical deformity.

We also looked at characterizing how the progression of cervical deformity leads to compensatory changes in the thoracic and lumbar spine. We identified 18 patients who exclusively had cervical anomalies and correlated the magnitude of the deformity to other radiographic parameters. By isolating these patients, we theorized that any changes manifesting along the thoracic or lumbar spines would be primarily driven by cervical deformity, as opposed to being driven by intrinsic vertebral anomalies within the thoracic or lumbar spines. Our key findings in this analysis were that the cervical coronal curve angle positively correlated with the lumbar coronal curve (ρ = 0.403; *p* = 0.037) and coronal balance, as measured by C2 (ρ = 0.407, *p* = 0.035) and C7-CSVL (ρ = 0.403; *p* = 0.037), but not with the compensatory thoracic curve (ρ = 0.218; *p* = 0.247). Moreover, we found a significant negative correlation between the cervical curve and T1S. The compensatory lumbar curve also positively correlated with the compensatory thoracic curve (*p* = 0.048), although the correlation was more robust with the cervical curve (ρ = 0.409) than with the thoracic curve (ρ = 0.377).

These results suggest that as the cervical deformity worsens, the lumbar spine compensates more to offset the increased coronal imbalance. One reason we may be seeing this effect is that the lumbar spine is especially flexible at young ages [18], and patients with significant coronal deformity likely maintain some degree of coronal balance more through compensatory changes in the lumbar spine than in the thoracic spine. As the cervical coronal curve in CCS worsens, the lumbar region compensates first. However, as the patient ages and coronal curves worsen further, there may eventually be increased compensation in the thoracic region as well.

Overall, our results indicate that progression of cervical deformity in CCS is most closely associated with specific radiographic parameters indicating global spinal imbalance: C2-CSVL, C7-CSVL, and T1S ≥ 25°. While indications for surgery in this population are largely subjective to clinical judgment and individual surgeons’ biases, some groups such as Yu et al. [9] posit that clinically evident deformity or oblique head slope are surgical indications, and others [3,19,20] recommend operative intervention in patients < 5 years of age with unilateral bars due to their overall poor prognoses. Our study suggests that patients who present with T1S ≥ 25° may be appropriate surgical candidates, while the positive correlation of cervical deformity with C2-CSVL and C7-CSVL likely reflects parallel markers for worse cervical deformity. As such, we suggest thorough study of each patient’s spine on AP/lateral radiographs, with special attention paid to T1S, C2-CSVL, C7-CSVL, and the lumbar curve. It is possible that two-dimensional imaging may be insufficient and that three-dimensional imaging may allow for better understanding of the full complexity of a patient’s deformity. The lumbar curve may play a greater role in maintaining overall balance in CCS than previously thought, and worsening of the lumbar curve may be a proxy indicator for worsening cervical deformity as well. Further investigation into operative and nonoperative management of pediatric CCS is warranted, and our parameters may assist provider decision making with respect to this unique patient population.

This study’s limitations include small sample sizes with respect to our cohort, which limits the power of our statistical analysis. Furthermore, the retrospective nature of this study presents inherent limitations, and prospective studies in the future would provide more robust findings and conclusions that can guide clinical decision-making. Lastly, our study was unable to correlate clinical and radiographic spinal deformity with quality-of-life measures. Improving our understanding of how cervical deformity affects the patients’ quality of life pre- and postoperatively would provide us with greater knowledge with which to guide the decision to pursue surgical intervention.

## 5. Conclusions

CCS is an extremely rare but challenging disease to manage. Overall surgical indications are unclear, but this present study suggests that T1S ≥ 25° may serve as a useful marker to assess risk of progression. Operative intervention may be appropriate for these patients. Greater scrutiny should also be paid towards compensatory lumbar curves, as our results suggest that the lumbar spine curve worsens as the cervical deformity progresses. Our series presents one of the larger reports on the treatment of CCS, which is an extremely rare condition that is difficult to treat. The limitations of our study were the small sample size and retrospective design.

## Figures and Tables

**Table 1:** List of associated primary diagnoses at the time of presentation with frequency and sex distribution within this series.

	Total Number (%)	Number of Boys (%)
Klippel–Feil syndrome	23 (60)	11 (48)
Torticollis	8 (21)	6 (75)
VACTERL syndrome	4 (10)	1 (25)
Tethered cord syndrome	3 (7.5)	1 (33)
Goldenhar syndrome	3 (7.5)	1 (33)
Sprengel’s deformity	3 (7.5)	0 (0)
Larsen syndrome	2 (5)	1 (50)
Osteogenesis imperfecta	1 (2.5)	1 (100)
Brown syndrome	1 (2.5)	1 (100)
Bruck syndrome	1 (2.5)	1 (100)
Poland’s syndrome	1 (2.5)	0 (0)
Total	38 (100)	22 (100)

**Table 2:** Mean and SD for subgroups of children with and without progression.

	At Presentation			Last Follow-Up			Interval Change		
	No Progression (N = 22)	Progression (N = 16)	*p*-Value	No Progression (N = 22)	Progression (N = 16)	*p*-Value	No Progression (N = 22)	Progression (N = 16)	*p*-Value
Age (yr)	5.8 ± 3.9	5.4 ± 4.5	0.801	8.8 ± 3.3	8.5 ± 4.4	0.859	3.2 ± 3.7	2.8 ± 1.8	0.725
Weight (kg)	18.7 ± 8.4	18.2 ± 11.3	0.893	25.9 ± 11.7	26.7 ± 13.1	0.829	7.7 ± 11.4	5.8 ± 6.1	0.558
Height (cm)	103.3 ± 16.7	98.5 ± 24.5	0.534	122.7 ± 19.2	120.9 ± 23.9	0.867	21.5 ± 30.6	18.7 ± 12.4	0.771
Cervical coronal curve (°)	18.6 ± 15.8	16.3 ± 18.1	0.682	22.2 ± 15.8	26.2 ± 18.4	0.520	5.3 ± 4.0	8.4 ± 7.2	0.151
Thoracic coronal curve (°)	30.2 ± 24.0	27.0 ± 18.3	0.743	54.1 ± 22.6	33.2 ± 20.6	0.063	12.9 ± 16.5	7.9 ± 9.9	0.436
Lumbar coronal curve (°)	27.7 ± 14.3	19.1 ± 9.6	0.225	39.7 ± 19.8	26.6 ± 8.9	0.158	11.7 ± 7.9	5.8 ± 15.4	0.502
C2 CSVL (mm)	24.4 ± 11.1	6.9 ± 25.2	0.860	18.2 ± 25.8	17.0 ± 23.4	0.921	18.4 ± 23.1	−4.9 ± 21.8	0.069
C7 CSVL (mm)	25.4 ± 17.1	6.9 ± 36.3	0.200	20.6 ± 24.9	15.5 ± 32.7	0.739	18.7 ± 48.8	−2.9 ± 13.7	0.224
C2 SVA (mm)	57.5 ± 41.1	20.8 ± 30.7	0.053	38.0 ± 10.9	28.0 ± 39.7	0.569	−11.2 ± 64.5	11.5 ± 47.0	0.495
C7 SVA (mm)	51.3 ± 35.8	20.9 ± 29.7	0.075	20.2 ± 4.5	25.0 ± 43.4	0.813	−9.6 ± 67.7	3.0 ± 52.6	0.723
Thoracic kyphosis (°)	40.0 ± 12.5	44.0 ± 12.4	0.476	36.0 ± 4.8	43.7 ± 15.0	0.279	−2.0 ± 10.6	0.9 ± 8.7	0.551
Lumbar lordosis (°)	43.5 ± 13.6	50.4 ± 11.9	0.233	45.3 ± 12.1	59.3 ± 13.2	0.056	8.8 ± 13.0	11.7 ± 15.2	0.687
T1 slope (°)	23.8 ± 7.1	32.4 ± 14.0	0.041	25.4 ± 6.9	26.2 ± 8.3	0.784	−1.1 ± 8.9	−2.5 ± 13.1	0.741
Cervical kyphosis (°)	−7.3 ± 20.1	−10.4 ± 21.3	0.666	−7.9 ± 26.0	−9.5 ± 24.1	0.859	1.6 ± 26.1	−3.6 ± 12.0	0.508
T1S–cervical kyphosis (°)	29.6 ± 24.8	42.8 ±30.1	0.164	33.4 ± 29.3	35.7 ± 27.8	0.819	0.9 ± 20.4	1.1 ± 18.4	0.978
Occiput-C2 (°)	25.5 ± 14.2	29.3 ± 16.4	0.470	18.8 ± 9.8	20.4 ± 15.0	0.714	−7.4 ± 14.9	−4.7 ± 11.7	0.598
C2-T3 (°)	16.2 ± 11.4	21.9 ± 16.7	0.238	17.1 ± 16.0	19.9 ± 15.9	0.624	−2.9 ± 10.7	2.3 ± 10.2	0.201

**Table 3:** Progression of scoliosis variables from presentation to last follow-up.

Variable	Presentation	Last Follow-Up	Δ	Significance
Age (years)	7.0 ± 3.9	8.7 ± 3.9	1.5 ± 1.5	0.004
Height (cm)	108.8 ± 15.1	120.1 ± 17.4	8.9 ± 8.7	0.016
Weight (kg)	21.2 ± 10.1	24.3 ± 10.2	2.6 ± 3.4	0.021
Cervical major coronal curve (°)	16.0 ± 14.7	21.7 ± 16.4	5.7 ± 5.6	0.005
Thoracic major coronal curve (°)	33.7 ± 25.6	34.6 ± 24.4	2.7 ± 15.4	0.640
Lumbar major coronal curve (°)	14.8 ± 10.0	15.5 ± 9.5	1.4 ± 9.8	0.673
C2-CSVL (mm)	20.2 ± 15.8	14.0 ± 15.7	−2.0 ± 23.9	0.817
C7-CSVL (mm)	22.9 ± 19.0	15.6 ± 15.1	−3.7 ± 18.7	0.597
C2-SVA (mm)	34.7 ± 48.8	40.4 ± 47.6	18.1 ± 67.6	0.582
C7-SVA (mm)	29.5 ± 42.8	27.7 ± 52.6	15.8 ± 66.2	0.585
Thoracic kyphosis (°)	43.5 ± 10.6	38.0 ± 7.2	0.3 ± 7.5	0.924
Lumbar lordosis (°)	43.8 ± 16.6	48.0 ± 17.4	4.0 ± 3.8	0.040
T1 slope (°)	23.5 ± 8.5	23.1 ± 5.4	0.8 ± 7.4	0.714
T1S-CL (°)	29.9 ± 29.8	24.8 ± 34.3	0.6 ± 17.4	0.642
Cervical lordosis (°)	−8.1 ± 23.5	−1.7 ± 33.1	4.9 ± 28.0	0.540
O-C2 angle (°)	27.2 ± 15.9	22.1 ± 14.9	−2.1 ± 10.4	0.484
C2-T3 angle (°)	14.7 ± 10.3	17.7 ± 16.5	3.3 ± 11.0	0.324

cm = centimeters; kg = kilograms; C2 = cervical 2; CSVL = central sacral vertical line; mm = millimeters; C7 = cervical 7; SVA = sagittal–vertical axis; T1S = Thoracic 1 slope; CL = cervical lordosis; O = occiput.

**Table 4:** Comparison of thoracic and cervical major curve Pearson correlations with other scoliosis variables.

Variable	ρ _Cervical major curve_	Significance	ρ _Thoracic major curve_	Significance
Age (years)	−0.021	0.899	0.031	0.873
Height (cm)	−0.275	0.128	−0.048	0.811
Weight (kg)	−0.312	0.068	−0.082	0.672
Cervical major coronal curve (°)			0.218	0.247
Thoracic major coronal curve (°)	0.218	0.247		
Lumbar major coronal curve (°)	0.409	0.028	0.377	0.048
C2-CSVL (mm)	0.407	0.035	−0.105	0.609
C7-CSVL (mm)	0.403	0.037	−0.067	0.744
C2-SVA (mm)	−0.185	0.398	0.177	0.432
C7-SVA (mm)	−0.121	0.565	−0.033	0.882
Thoracic kyphosis (°)	0.083	0.699	−0.402	0.063
Lumbar lordosis (°)	−0.172	0.411	−0.437	0.037
T1 slope (°)	−0.344	0.034	−0.135	0.493
T1S-CL (°)	−0.108	0.519	−0.537	0.003
Cervical lordosis (°)	0.009	0.955	0.408	0.028
O-C2 angle (°)	−0.002	0.993	−0.128	0.51
C2-T3 angle (°)	−0.011	0.95	−0.267	0.177

## Data Availability

The datasets generated during and/or analyzed during the current study are not publicly available, but are available from the corresponding author on reasonable request.
